# Supplementary material for: A reductionist paradigm for high-throughput behavioural fingerprinting in Drosophila melanogaster
Source: eLife. 2023 Nov 8;12:RP86695. doi: 10.7554/eLife.86695 (PMC10631757; doi:10.7554/eLife.86695)
Supplement: Supplementary file 3 [file elife-86695-supp1.zip › SupplementaryTable1.odt]

| Compound | Mode of action | Ref | Figure |
| --- | --- | --- | --- |
| Syngenta Cpd 3 | Mitochondrial ATP synthesis |  | 1D |
| DDT | Voltage-gated sodium channel activator | 1 | 1C |
| Juglone | ROS | 2,3 | 1D |
| Diflubenzuron | Chitin synthesis | 4,5 | 1D |
| Rotenone | Complex I of the mitochondrial respiratory chain (MRC) | 6 | 1D |
| Methotrexate | Dihydrofolate reductase inhibitor | 7 | 1D |
| Diafenthiuron | Mitochondrial ATPase | 8 | 1D |
| Fenoxycarb | Juvenile hormone mimic | 9 | 1D |
| DCCD | Mitochondrial FIFO ATP synthase | 10 | 1D |
| Lufenuron | Chitin synthesis inhibitor | 11 | 1C |
| Chlorfenapyr | Mitochondrial oxidative phosphorylation | 12 | 1D |
| Thiamethoxam | NACh-R13 | 13 | 1D |
| Tebfenozide | Ecdysone receptor | 14 | 1D |
| Flonicamid | Sodium channels/TRP-V channels | 15 | 1C |
| Syngenta Cpd 2 | NACh-R16 | 16 | 1D |
| Quinuclidine Hycl. | Muscarinic Ach-R | 17 | 1D |
| Indoxacarb | Voltage-gated sodium channels | 18 | 1D |
| Fluazinam | Mitochondrial ATP synthesis | 19,20 | 1D |
| Syngenta Cpd 5 | Unknown |  | 1D |
| Syngenta Cpd 7 | Octopamine receptor |  | 1D |
| Methoprene | Juvenile hormone mimic | 21,22 | 1D |
| Permethrin | Voltage-gated sodium channels | 23 | 1C |
| Tubulin | Unknown |  | 1D |
| Metaflumizone | Voltage-gated sodium channels | 24 | 1D |
| Syngenta Cpd 12 | Unknown |  | 1D |
| Cyenopyrafen | Succinate dehydrogenase | 25,26 | 1D |
| Syngenta Cpd 1 | GABAA receptors |  | 1C |
| Syngenta Cpd 10 | Octopamine receptor | 27 | 1D |
| Abamectin | Glutamate-gated chloride channels | 28,29 | 1C,1D,2 |
| Diamide Syn Cpd | Ryanodine receptors |  | 1C,1D,2 |
| Chlorantraniliprole | Ryanodine receptors | 30 | 1C,1D,2 |
| Cyantraniliprole | Ryanodine receptors | 31 | 1C,1D,2 |
| Dieldrin | GABAA receptors | 32 | 1C,1D,2 |
| Flubendiamide | Ryanodine receptors | 33 | 1C,1D,2 |
| Imidacloprid | NACh-R | 34,35 | 1C,1D,2 |
| Pymetrozine | TRP-V channels | 36 | 1C,1D,2 |
| Ryanodine | Ryanodine receptors | 37 | 1C,1D,2 |
| Spiroindoline | Cholinergic transmission | 38 | 1C,1D,2 |
| Tetramethrin | Sodium channel modulator | 39 | 1C,1D,2 |

Bibliography for references listed in supplementary table 1 only (see column “Ref”).

1.	O’Brien, R. D. & Matsumura, F. DDT: A New Hypothesis of Its Mode of Action. Science 146, 657–658 (1964).

2.	Islam, A. K. M. M. & Widhalm, J. R. Agricultural Uses of Juglone: Opportunities and Challenges. Agronomy 10, 1500 (2020).

3.	Maleita, C. et al. Juglone and 1,4-Naphthoquinone—Promising Nematicides for Sustainable Control of the Root Knot Nematode Meloidogyne luci. Front. Plant Sci. 13, 867803 (2022).

4.	Cohen, E. & Casida, J. E. Inhibition of Tribolium gut chitin synthetase. Pestic. Biochem. Physiol. 13, 129–136 (1980).

5.	Meola, S. M. & Mayer, R. T. Inhibition of Cellular Proliferation of Imaginal Epidermal Cells by Diflubenzuron in Pupae of the Stable Fly. Science 207, 985–987 (1980).

6.	Palmer, G., Horgan, D. J., Tisdale, H., Singer, T. P. & Beinert, H. Studies on the Respiratory Chain-linked Reduced Nicotinamide Adenine Dinucleotide Dehydrogenase. J. Biol. Chem. 243, 844–847 (1968).

7.	Stone, S. R., Montgomery, J. A. & Morrison, J. F. Inhibition of dihydrofolate reductase from bacterial and vertebrate sources by folate, aminopterin, methotrexate and their 5-deaza analogues. Biochem. Pharmacol. 33, 175–179 (1984).

8.	Ruder, F. J. & Kayser, H. The Carbodiimide Product of Diafenthiuron Inhibits Mitochondria in Vivo. Pestic. Biochem. Physiol. 46, 96–106 (1993).

9.	Grenier, S. & Grenier, A.-M. Fenoxycarb, a fairly new Insect Growth Regulato: review of its effects on insects. Ann. Appl. Biol. 122, 369–403 (1993).

10.	Ruder, F. & Kayser, H. The thiourea insecticide diafenthiuron inhibits mitochondrial ATPase in vitro and in vivo by its carbodiimide product. Biochem. Soc. Trans. 22, 241–244 (1994).

11.	Wilson, T. G. & Cryan, J. R. Lufenuron, a chitin-synthesis inhibitor, interrupts development ofDrosophila melanogaster. J. Exp. Zool. 278, 37–44 (1997).

12.	Black, B. C., Hollingworth, R. M., Ahammadsahib, K. I., Kukel, C. D. & Donovan, S. Insecticidal Action and Mitochondrial Uncoupling Activity of AC-303,630 and Related Halogenated Pyrroles. Pestic. Biochem. Physiol. 50, 115–128 (1994).

13.	Wiesner, P. & Kayser, H. Characterization of nicotinic acetylcholine receptors from the insectsAphis craccivora,Myzus persicae, andLocusta migratoria by radioligand binding assays: Relation to thiamethoxam action. J. Biochem. Mol. Toxicol. 14, 221–230 (2000).

14.	Retnakaran, A. et al. Mode of action of the ecdysone agonist tebufenozide (RH-5992), and an exclusion mechanism to explain resistance to it. Pest Manag. Sci. 57, 951–957 (2001).

15.	Taylor-Wells, J. et al. Toxicity, mode of action, and synergist potential of flonicamid against mosquitoes. Pestic. Biochem. Physiol. 151, 3–9 (2018).

16.	Chambers, C. et al. Insecticidal spider toxins are high affinity positive allosteric modulators of the nicotinic acetylcholine receptor. FEBS Lett. 593, 1336–1350 (2019).

17.	Dick, M. R., Dripps, J. E. & Orr, N. Muscarinic Agonists as Insecticides and Acaricides. Pestic. Sci. 49, 268–276 (1997).

18.	Lapied, B., Grolleau, F. & Sattelle, D. B. Indoxacarb, an oxadiazine insecticide, blocks insect neuronal sodium channels: DCJW blocks neuronal Na + channels. Br. J. Pharmacol. 132, 587–595 (2001).

19.	Lee, J. E. et al. Fluazinam targets mitochondrial complex I to induce reactive oxygen species-dependent cytotoxicity in SH-SY5Y cells. Neurochem. Int. 60, 773–781 (2012).

20.	Guo, Z., Miyoshi, H., Komyoji, T., Haga, T. & Fujita, T. Uncoupling activity of a newly developed fungicide, fluazinam [3-chloro-N-(3-chloro-2,6-dinitro-4-trifluoromethylphenyl)-5-trifluoromethyl-2-pyridinamine]. Biochim. Biophys. Acta BBA - Bioenerg. 1056, 89–92 (1991).

21.	Miura, K., Oda, M., Makita, S. & Chinzei, Y. Characterization of the Drosophila Methoprene -tolerant gene product: Juvenile hormone binding and ligand-dependent gene regulation. FEBS J. 272, 1169–1178 (2005).

22.	Jindra, M., Palli, S. R. & Riddiford, L. M. The Juvenile Hormone Signaling Pathway in Insect Development. Annu. Rev. Entomol. 58, 181–204 (2013).

23.	Vais, H., Williamson, M. S., Devonshire, A. L. & Usherwood, P. N. The molecular interactions of pyrethroid insecticides with insect and mammalian sodium channels. Pest Manag. Sci. 57, 877–888 (2001).

24.	Salgado, V. L. & Hayashi, J. H. Metaflumizone is a novel sodium channel blocker insecticide. Vet. Parasitol. 150, 182–189 (2007).

25.	Nakahira, K. Strategy for discovery of a novel miticide Cyenopyrafen which is one of electron transport chain inhibitors. J. Pestic. Sci. 36, 511–515 (2011).

26.	Wei, P. et al. Downregulation of carboxylesterase contributes to cyflumetofen resistance in Tetranychus cinnabarinus (Boisduval): Downregulated esterase genes in cyflumetofen resistance in mites. Pest Manag. Sci. 75, 2166–2173 (2019).

27.	O’Sullivan, A. C. et al. Synthesis and Insecticidal Activity of New Benzyl- and Indanyl-Oxazolines, Thiazolines and Alkoxy-Alkyl-Imidazolines. in ACS Symposium Series (eds. Maienfisch, P. & Stevenson, T. M.) vol. 1204 411–430 (American Chemical Society, 2015).

28.	Cully, D. F., Paress, P. S., Liu, K. K., Schaeffer, J. M. & Arena, J. P. Identification of a Drosophila melanogaster Glutamate-gated Chloride Channel Sensitive to the Antiparasitic Agent Avermectin. J. Biol. Chem. 271, 20187–20191 (1996).

29.	Arena, J. P., Liu, K. K., Paress, P. S., Schaeffer, J. M. & Cully, D. F. Expression of a glutamate-activated chloride current in Xenopus oocytes injected with Caenorhabditis elegans RNA: evidence for modulation by avermectin. Mol. Brain Res. 15, 339–348 (1992).

30.	Cordova, D. et al. Anthranilic diamides: A new class of insecticides with a novel mode of action, ryanodine receptor activation. Pestic. Biochem. Physiol. 84, 196–214 (2006).

31.	Selby, T. P. et al. Discovery of cyantraniliprole, a potent and selective anthranilic diamide ryanodine receptor activator with cross-spectrum insecticidal activity. Bioorg. Med. Chem. Lett. 23, 6341–6345 (2013).

32.	ffrench-Constant, R. H., Rocheleau, T. A., Steichen, J. C. & Chalmers, A. E. A point mutation in a Drosophila GABA receptor confers insecticide resistance. Nature 363, 449–451 (1993).

33.	Masaki, T. et al. Flubendiamide, a Novel Ca 2+ Channel Modulator, Reveals Evidence for Functional Cooperation between Ca 2+ Pumps and Ca 2+ Release. Mol. Pharmacol. 69, 1733–1739 (2006).

34.	Bodereau-Dubois, B. et al. Transmembrane Potential Polarization, Calcium Influx, and Receptor Conformational State Modulate the Sensitivity of the Imidacloprid-Insensitive Neuronal Insect Nicotinic Acetylcholine Receptor to Neonicotinoid Insecticides. J. Pharmacol. Exp. Ther. 341, 326–339 (2012).

35.	Perry, T. et al. Effects of mutations in Drosophila nicotinic acetylcholine receptor subunits on sensitivity to insecticides targeting nicotinic acetylcholine receptors. Pestic. Biochem. Physiol. 102, 56–60 (2012).

36.	Nesterov, A. et al. TRP Channels in Insect Stretch Receptors as Insecticide Targets. Neuron 86, 665–671 (2015).

37.	Fleischer, S., Ogunbunmi, E. M., Dixon, M. C. & Fleer, E. A. Localization of Ca2+ release channels with ryanodine in junctional terminal cisternae of sarcoplasmic reticulum of fast skeletal muscle. Proc. Natl. Acad. Sci. 82, 7256–7259 (1985).

38.	Sluder, A. et al. Spiroindolines Identify the Vesicular Acetylcholine Transporter as a Novel Target for Insecticide Action. PLoS ONE 7, e34712 (2012).

39.	Yamamoto, D., Quandt, F. N. & Narahashi, T. Modification of single sodium channels by the insecticide tetramethrin. Brain Res. 274, 344–349 (1983).
